# Factors associated with the uptake of newly introduced childhood vaccinations in Ethiopia: the cases of rotavirus and pneumococcal conjugate vaccines

**DOI:** 10.1186/s12889-019-8002-8

**Published:** 2019-12-10

**Authors:** Abrham Wondimu, Qi Cao, Jan C. Wilschut, Maarten J. Postma

**Affiliations:** 1 Department of Pharmaceutics, School of Pharmacy, College of Medicine and Health Sciences, University of Gondar, Gondar, Ethiopia; 2 Unit of PharmacoTherapy, ­Epidemiology & ­Economics (PTE2), Department of Pharmacy, University of Groningen, Groningen, The Netherlands; 3 Department of Medical Microbiology, University Medical Center Groningen (UMCG), University of Groningen, Groningen, The Netherlands; 4 Department of Health Sciences, University Medical Center Groningen (UMCG), University of Groningen, Groningen, The Netherlands; 5 Department of Economics, Econometrics & Finance, Faculty of Economics & Business, University of Groningen, Groningen, The Netherlands

**Keywords:** Childhood immunization, Rotavirus vaccine, Pneumococcal conjugate vaccine, Vaccine uptake, Vaccination status, Ethiopia

## Abstract

**Background:**

Childhood immunization programmes have made substantial contributions to lowering the burden of disease among children in developing countries, however a large proportion of children still remain unimmunized. This study aimed to explore the determinants of rotavirus vaccine (RVV) and pneumococcal conjugate vaccine (PCV) uptake in Ethiopia**.**

**Methods:**

The 2016 Ethiopian demographic and health survey dataset was used in this analysis. A total of 2004 children aged 12–23 months were included in the analysis. A multivariable logistic regression model was employed to identify the determinants of uptake of the complete schedules of RVV (two doses) and PCV (three doses). Crude and adjusted odds ratios with 95% confidence intervals (CIs) were calculated.

**Results:**

The uptakes of the complete schedules of RVV and PCV among children aged 12–23 months were 56 and 49.1%, respectively. The likelihood of immunization with the complete schedule of RVV was significantly lower among children from the relatively poor Afar region in Ethiopia (AOR 0.16; 95%-CI 0.04–0.61). Similarly, children living in not only the Afar region (AOR 0.10; 95%-CI 0.03–0.38), but also the Gambela region (AOR 0.25; 95%-CI 0.08–0.83), were less likely to be vaccinated with PCV. On the other hand, children from more wealthy households had higher odds of vaccination with RVV (AOR 1.69; 95%-CI 1.04–2.75). Also attending antenatal care (ANC) was found to be significantly associated with uptake of the complete schedule of RVV and PCV.

**Conclusions:**

The uptake of RVV and PCV is suboptimal in Ethiopia. The uptake of the vaccines were found to be associated with region, ANC use and wealth status.

## Background

Diarrhea and pneumonia are the cause of heavy health burdens across the world [1, 2]. Globally, in 2011, diarrhea and pneumonia were respectively responsible for the death of 0.7 and 1.3 million children under the age of five [1]. In Ethiopia, diarrhoea and pneumonia are the two leading causes of morbidity and mortality among children in this age group [3, 4]. In addition, a considerable economic burden was reported in Ethiopia due to the extensive household expenditures of treating diarrhoea or pneumonia [5]. In the absence of targeted immunization programmes, rotavirus is associated with 28% of severe diarrheal cases and *Streptococcus pneumoniae* is responsible for 18% of cases of severe pneumonia [1].

The effectiveness of rotavirus vaccines (RVVs) and pneumococcal conjugate vaccines (PCVs) in preventing gastroenteritis and pneumonia has been established [6, 7]. The World Health Organization (WHO) recommended the introduction of these vaccines into the immunization schedules of developing countries with high background rates of childhood mortality [8, 9]. Following the recommendation, the Federal Ministry of Health (FMOH) of Ethiopia introduced into the routine childhood immunization schedule the 10-valent PCV (PCV10) in 2011 and the RVV in 2013 with financial support from the GAVI Alliance [10].

The uptake of a vaccine is one of the key performance indicators of immunization programmes [11]. According to the Global Vaccine Action Plan (GVAP)—which was endorsed at the 65th World Health Assembly in May 2012— all countries should plan to reach 90% national coverage of three doses of diphtheria-pertussis-tetanus (DTP) vaccines by 2015 and all vaccines in national vaccination programmes by 2020 [12]. However, the WHO’s report indicated that global immunization coverage was only at 85% in 2017, and 19.9 million children did not receive routine life-saving vaccinations in that same year. The report also showed that the coverage level for new and underused vaccines remains low only 28% for RVV and 44% for PCV [13].

Despite the government’s improvement measures in Ethiopia—such as the Reach Every District (RED) approach and health extension programme—full immunization coverage in the country remained very low at 33.3% for all age-appropriate vaccinations, including three doses of PCV and two doses of RVV. In addition, there is marked heterogeneity of the vaccination coverage across different regions of the country [10, 14–16]. In Ethiopia, studies that were conducted to identify factors associated with immunization coverage among children commonly focused on full immunization coverage [17–19]. Few studies have also investigated determinants of coverage for specific vaccines included in the national expanded programme on immunization (EPI) [20, 21]. So far, however, very little attention has been paid to newly introduced vaccines such as RVV and PCV. Therefore, this study aims to investigate the factors associated with uptake of RVV and PCV in Ethiopia.

## Methods

### Study design

A secondary analysis of the 2016 Ethiopian Demographic and Health Survey (EDHS) data was conducted. The EDHS is a population-based cross-sectional household survey which was conducted in nine regions (Tigray, Afar, Amhara, Oromiya, Somali, Benishangul Gumuz, Southern Nations Nationalities and Peoples (SNNP), Gambela, and Harari) and two administrative cities (Addis Ababa and Dire Dawa) in Ethiopia. The detailed study design of the EDHS was described elsewhere [15].

### Sampling technique

The 2016 EDHS employed a stratified sampling technique to recruit study participants. Each region was stratified into urban and rural areas, yielding 21 sampling strata. In the first stage, a total of 645 clusters were selected from urban (202 clusters) and rural (443 clusters) areas with a sampling probability proportional to the population size. Then households in each of the selected clusters were listed and used as a sampling frame for the selection of households. In the second stage, a fixed number of 28 households in each cluster were selected by equal probability systematic sampling in the selected cluster. The final survey was conducted in 5232 and 11,418 residential households in urban and rural areas, respectively. All women between 15 and 49 years of age, who were either permanent residents of the selected households or visitors who stayed in the household the night before the survey, were eligible to be interviewed. Accordingly, complete interviews were conducted with 15,683 women, corresponding to a response rate of about 95% [15].

### Data collection

Data on the vaccination status of the children was collected from the mother’s report and/or vaccination card (this also includes a booklet or other home-based record). If the card was available at home or health facility, information about the vaccination status was directly obtained from the vaccination card. Otherwise, the mother was asked to recall all vaccinations received by their children. According to the 2016 EDHS, data collectors were able to see a vaccination card at home for 34.1% of children aged 12–23 months [15].

### Study variables

The vaccination statuses for RVV and PCV for children aged 12–23 months were the outcome variables. Children in this age group are expected to have received the complete schedule of these vaccines, according to the EPI schedule in Ethiopia (Table 1). The vaccination status was considered separately for the two vaccines. A child who received two doses of RVV and three doses of PCV was considered “fully vaccinated” with the respective vaccines whereas a child who did not receive at least one dose of the vaccines was categorized as “not (fully) vaccinated”. The independent variables included in this study were the following:

- Mother’s characteristics, including age at child birth, education level, marital status, occupation, religion, region, residence place, exposure to media (i.e. access to newspaper, radio and television; those who had access to any of these three outlets at least once a week were considered as exposed to media and others were considered not exposed), use of ANC (≥ 4 ANC visits was considered as adequate, whereas 1–3 visits was considered as inadequate [22]), place of delivery, postnatal check-up and the perception that the distance to a health facility was a big problem.
- Partner’s level of education and occupation.
- Child’s characteristics including sex and birth order.
- Household wealth index, a composite measure of the socioeconomic status of the household used for ranking the households from poorest to richest quantiles. It is measured based on assets ownership and housing characteristics including a source of drinking water, type of toilet facilities, type of cooking fuel, materials used for housing construction, ownership of land, and other assets.

**Table 1:** National EPI schedule by antigens in Ethiopia

Schedule	Antigens
Birth	BCG, OPV0
6 weeks	PENTA1, OPV1, PCV1, RVV1
10 weeks	PENTA2, OPV2, PCV2, RVV2
14 weeks	PENTA3, OPV3, PCV3, IPV
9 months	Measles

*EPI* Expanded programme on immunization, *BCG* Baccilus Calmette-Guerin, *OPV* Oral Polio Vaccine, *PENTA* Pentavalent Vaccine (Diptheria-Pertussis-Tetanus, Hepatitis B *Haemophilus Influenzae* type B), *PCV* Pneumococcal Conjugate Vaccine, *RVV* Rotavirus Vaccine, *IPV*: Inactivated Polio Vaccine

### Analysis

Stata 15.1 was used for data analysis (College Station, TX: StataCorp LLC). Descriptive statistics were employed to determine the distribution of independent variables by vaccination status. A chi-square test was used to test whether there were associations between vaccination status and background characteristics. Bivariate analysis was used to separately evaluate the effect of each independent variable on the outcome variable and the variables with a *p*-value <0.25 in the bivariate analysis were included in the multivariate logistic regression models [23]. Child’s sex as a demographic characteristic was added in the final model [24]. Variance Inflation Factor (VIF) was employed to detect the presence of multicollinearity among independent variables at the cutoff point of 10 [25]. As a result, partner occupation with VIF greater than 10 was omitted from the multivariate logistic regression models. An F-adjusted mean residual goodness-of-fit test was utilized to check the goodness-of-fit in the final model [26]. Due to the survey sample designs employed in DHS, *svyset* command in Stata 15.1 was used in order to account for inverse probability weights [15, 27].

## Results

In this study, the vaccination status of 2004 Ethiopian children was assessed. More than half (53.8%) of the children were female, slightly over one-third (36.6%) were delivered in health facilities, and 71.8% of all children were born within the first five in their birth order. The vast majority of the children were rural dwellers (88.4%), and their mothers were between 20 and 34 years of age (73.2%). Only 8.5% of the children had a mother who attended secondary or higher education and 45.7% of the children had a mother who was involved in some kind of work. In terms of religion, about two-fifths (39.2%) of the children had a Muslim mother, followed by Orthodox Christian (34.8%), Protestant (22.2%) or other (3.8%). Of the children, 25.2, 19.8, 22.4, 18.3, and 14.4% lived in the poorest, poorer, middle, richer, and richest households, respectively. More than 62% of the children belonged to mothers who attended at least one ANC service during their pregnancy (Table 2).

**Table 2:** RVV and PCV Immunization status by parental and child characteristics in Ethiopia

Variables	Number of Children (%)*^, a^	Fully vaccinated with RVV		Fully vaccinated with PCV	
		n (%)*	*P*-value**	n (%)*	*P*-value**
Mother’s age at birth					
< 20	84 (4.2)	44 (52.5)	0.639	36 (42.2)	0.328
20–34	1467 (73.2)	836 (57.0)		741 (50.5)	
35–49	453 (22.6)	242 (53.4)		208 (45.9)	
Mother’s education level					
No education	1257 (62.7)	624 (49.6)	<0.001	533 (42.4)	<0.001
Primary	577 (28.8)	361 (62.7)		329 (57.1)	
Secondary and Higher	170 (8.5)	136 (80.1)		122 (71.7)	
Religion					
Orthodox	697 (34.8)	454 (65.1)	<0.001	435 (62.4)	<0.001
Protestant	445 (22.2)	258 (57.9)		219 (49.1)	
Muslim	786 (39.2)	363 (46.2)		303 (38.5)	
Others	76 (3.8)	47 (62.2)		28 (36.5)	
Region					
Tigray	152 (7.6)	121 (79.8)	<0.001	118 (77.7)	<0.001
Afar	20 (1)	5 (23.3)		3 (17.5)	
Amhara	364 (18.2)	215 (59.1)		220 (60.5)	
Oromia	881 (44)	442 (50.2)		337 (38.3)	
Somali	76 (3.8)	31 (41.3)		26 (34.9)	
Benishangul	21 (1)	16 (76.6)		15 (71.0)	
SNNPR	419 (20.9)	229 (54.7)		204 (48.6)	
Gambela	5 (0.3)	3 (60.5)		2 (46.1)	
Harari	5 (0.2)	3 (61.3)		3 (58.6)	
Addis Ababa	52 (2.6)	48 (91.7)		48 (91.4)	
Dire Dawa	9 (0.5)	8 (85.3)		7 (75.3)	
Wealth index					
Poorest	504 (25.2)	220 (43.6)	<0.001	181 (36.0)	<0.001
Poorer	396(19.8)	201(50.7)		194(48.9)	
Middle	450(22.4)	238(53.0)		199(44.2)	
Richer	366(18.3)	237(64.6)		205(56.0)	
Richest	288(14.4)	226(78.6)		205(71.3)	
Marital status					
Never in union	16(0.8)	11(70.6)	0.586	8(47.6)	0.143
Married or living with a partner	1897(94.7)	1065(56.2)		944(49.8)	
Others	91(4.5)	45(49.6)		32(35.6)	
Maternal occupation					
Not working	1089(54.3)	576(52.9)	0.043	484(44.5)	0.005
Working	915(45.7)	546 (59.7)		500(54.6)	
Sex of child					
Male	926(46.2)	505(54.5)	0.444	450(48.6)	0.763
Female	1078(53.8)	617(57.2)		534(49.6)	
Birth order					
1	372(18.6)	241(64.7)	0.006	205(55.1)	0.020
2–5	1063(53)	598(56.3)		540(50.8)	
6+	569(28.4)	282(49.6)		239(42.0)	
Exposure to media					
Not exposed	1625(81.1)	851(52.4)	< 0.001	745(45.8)	< 0.001
Exposed	379(18.9)	270(71.3)		239(63.1)	
Use of antenatal care					
No ANC visit	712(37.4)	283(39.7)	< 0.001	204(28.7)	< 0.001
Inadequate ANC visit	561(29.4)	322(57.4)		300(53.6)	
Adequate ANC visit	632(33.2)	474(74.9)		437(69.2)	
Place of delivery					
Home	1271(63.4)	615(48.4)	< 0.001	506(39.8)	< 0.001
Health Facility	733(36.6)	506(69.1)		478(65.3)	
Place of Residence					
Rural	1772(88.4)	938(52.9)	0.002	815(46.0)	< 0.001
Urban	232(11.6)	184(79.1)		169(72.9)	
Distance to a health facility					
big problem	1201(59.9)	604(50.3)	< 0.001	525(43.8)	< 0.001
Not a big problem	803(40.1)	518(64.5)		459(57.1)	
Postnatal check-up					
No	1750(91.8)	956(54.6)	< 0.001	836(47.8)	0.003
Yes	155(8.2)	123(78.9)		105(67.8)	
Partner’s education level					
No education	899(47.6)	448(49.8)	< 0.001	382(42.5)	< 0.001
Primary	746(39.5)	433(58.1)		397(53.2)	
Secondary and Higher	245(12.9)	180(73.7)		161(65.9)	
Partner’s occupation					
Not working	158(8.4)	62(39.4)	0.011	56(35.2)	0.016
Working	1725(91.6)	993(57.6)		882(51.2)	
Number of children aged 0–5 years					
< =3	1970(98.3)	1102(55.9)	0.783	967(49.1)	0.862
> 3	34(1.7)	20(58.9)		17(51.1)	
Total	2004(100)	1122(56.0)		984(49.1)	

*Sampling weights were applied; ^a^total number varies between categories due to missing values; *RVV* Rotavirus vaccine, *PCV* Pneumococcal conjugate vaccine; **p-value calculated using χ2 analysis

It was found that 56 and 49.1% of the children received the complete schedule of RVV and PCV, respectively (Table 2, Additional file 1). Across regions, immunization coverage with the complete schedule of RVV ranged from 23.3% in Afar to 91.7% in Addis Ababa, and from 17.5% in Afar to 91.4% in Addis Ababa for PCV (Table 2). According to bivariate analysis, uptake of RVV and PCV were associated with various background characteristics (Table 3). Vaccination of children with complete schedules of RVV and PCV was significantly associated with educational level, religion, region, wealth quantile, and working status of the mothers. Similarly, vaccines uptake were associated with paternal educational and working status. Moreover, the likelihood of complete schedule uptake of the vaccines was higher among children of mothers who received ANC, gave birth in a health facility, or visited a health facility within 2 months after delivery for a postnatal check-up. The mother’s exposure to mass media was also positively associated with the uptake of RVV and PCV. The uptake of RVV and PCV was higher in urban areas compared to rural areas and the uptake of the vaccines was also higher among children of mothers who do not consider distance to a health facility as a big problem. The uptake of the complete schedule of both RVV and PCV did not significantly vary between male and female children.

**Table 3:** Factors associated with uptake of the complete schedules of RVV and PCV in children aged 12–23 months in Ethiopia, bivariate and multivariate regression analysis

Variables	PCV		RVV	
	COR (95% CI)	AOR (95% CI)	COR (95% CI)	AOR (95% CI)
Mother’s age at birth				
< 20	0.72(0.38–1.36)		0.84(0.42–1.66)	
20–34	1.00		1.00	
35–49	0.83(0.61–1.13)		0.86(0.61–1.22)	
Mother’s education level				
No education	1.00		1.00	
Primary	1.81(1.32–2.50)**	1.42(0.92–2.22)	1.71(1.26–2.31)*	1.35(0.91–2.00)
Secondary and Higher	3.45(1.72–6.90)**	1.00(0.33–3.05)	4.08(2.11–7.89)**	1.20(0.42–3.42)
Religion				
Orthodox	1.00		1.00	
Protestant	0.58(0.37–0.90)	0.83(0.47–1.46)	0.74(0.49–1.12)	0.97(0.54–1.76)
Muslim	0.38(0.25–0.56)**	0.68(0.42–1.12)	0.46(0.32–0.66)**	0.71(0.45–1.13)
Others	0.35(0.19–0.64)*	0.64(0.32–1.30)	0.88(0.53–1.48)	1.70(0.92–3.12)
Region				
Tigray	0.33(0.13–0.84)*	0.71(0.20–2.55)	0.36(0.14–0.92)*	1.06(0.28–3.99)
Afar	0.02(0.01–0.06)**	0.10(0.03–0.38)*	0.03(0.01–0.08)**	0.16(0.04–0.61)*
Amhara	0.14(0.06–0.35)**	0.59(0.16–2.23)	0.13(0.05–0.32)**	0.59(0.16–2.25)
Oromia	0.06(0.02–0.14)**	0.30(0.08–1.08)	0.09(0.04–0.22)**	0.58(0.16–2.21)
Somali	0.05(0.02–0.13)**	0.37(0.10–1.46)	0.06(0.02–0.16)**	0.58(0.15–2.22)
Benishangul	0.23(0.08–0.65)*	1.07(0.26–4.49)	0.30(0.11–0.77)*	1.86(0.45–7.69)
SNNPR	0.09(0.04–0.22)**	0.31(0.08–1.18)	0.11(0.05–0.26)**	0.45(0.11–1.83)
Gambela	0.08(0.03–0.21)**	0.25(0.08–0.84)*	0.14(0.05–0.36)**	0.45(0.13–1.59)
Harari	0.13(0.05–0.34)**	0.52(0.15–1.79)	0.14(0.05–0.37)**	0.66(0.18–2.43)
Addis Ababa	1.00		1.00	
Dire Dawa	0.29(0.10–0.79)*	0.71(0.19–2.61)	0.52(0.19–1.43)	1.94(0.48–7.85)
Wealth index				
Poorest	1.00		1.00	
Poorer	1.71(1.15–2.54)*	1.32(0.85–2.07)	1.33(0.86–2.06)	1.21(0.76–1.95)
Middle	1.41(0.95–2.10)	0.97(0.61–1.56)	1.46(0.92–2.30)	1.36(0.79–2.33)
Richer	2.27(1.49–3.46)**	1.29(0.76–2.19)	2.36(1.57–3.53)**	1.69(1.04–2.75)*
Richest	4.43(2.46–7.99)**	1.90(0.79–4.54)	4.75(2.52–8.95)**	1.84(0.75–4.50)
Marital status				
Never in union	1.00		1.00	
Married or living with a partner	1.09(0.30–4.02)		0.53(0.11–2.58)	
Others	0.61(0.15–2.48)		0.41(0.08–2.24)	
Maternal occupation				
Not working	1.00		1.00	
Working	1.50(1.13–2.00)*	1.04(0.75–1.44)	1.32(1.01–1.72)*	0.94(0.71–1.26)
Sex of child				
Male	1.00		1.00	
Female	1.04(0.80–1.37)	0.90(0.66–1.23)	1.12(0.84–1.49)	1.02(0.75–1.40)
Birth order				
1	1.00		1.00	
2–5	0.84(0.59–1.20)	1.26(0.80–2.01)	0.70(0.49–1.00)*	0.83(0.51–1.35)
6+	0.59(0.40–0.87)*	1.06(0.61–1.84)	0.54(0.37–0.78)*	0.77(0.45–1.33)
Exposure to media				
Not exposed	1.00		1.00	
Exposed	2.02(1.40–2.93)**	1.19(0.73–1.94)	2.26(1.50–3.42)**	1.38(0.80–2.38)
Use of antenatal care				
No ANC visit	1.00		1.00	
Inadequate ANC visit	2.87(1.91–4.32)**	2.20(1.43–3.37)**	2.04(1.39–3.01)**	1.64(1.06–2.53)*
Adequate ANC visit	5.59(3.77–8.30)**	3.27(2.04–5.23)**	4.54(3.05–6.74)**	2.79(1.73–4.51)**
Place of delivery				
Home	1.00		1.00	
Health Facility	2.84(2.08–3.88)**	1.41(0.97–2.06)	2.39(1.76–3.24)*	1.11(0.74–1.67)
Place of Residence				
Rural	1.00		1.00	
Urban	3.16(1.71–5.83)**	1.03(0.44–2.42)	3.36(1.54–7.34)*	1.29(0.40–4.18)
Distance to a health facility				
Big problem	1.00		1.00	
Not a big problem	1.71(1.30–2.25)**	0.92(0.66–1.28)	1.80(1.33–2.43)	1.14(0.80–1.63)
Postnatal check-up				
No	1.00		1.00	
Yes	2.30(1.32–4.00)*	1.01(0.56–1.81)	3.11(1.65–5.86)**	1.69(0.84–3.40)
Partner education				
No education	1.00		1.00	
Up to primary	1.54(1.14–2.08)*	1.31(0.88–1.95)	1.40(1.02–1.91)*	1.00(0.67–1.49)
Secondary and higher	2.61(1.63–4.18)**	0.91(0.44–1.87)	2.82(1.72–4.61)**	0.82(0.38–1.75)
Partner occupation^£^				
Not working	1.00		1.00	
Working	1.93(1.12–3.32)*		2.09(1.17–3.72)*	
Number of children aged 0–5 years				
< =3	1.00		1.00	
> 3	1.09(0.43–2.75)		1.13(0.47–2.71)	

*RVV* Rotavirus vaccine, *PCV* Pneumococcal conjugate vaccine, *COR* Crude odds ratio, *AOR* Adjusted odds ratio, *CI* Confidence Interval **P < 0.001), *p < 0.05, £: not included in the multivariate regression analysis due to collinearity

Table 3 also shows the results of the multivariate logistic regression. After adjusting for other variables, factors that remained significant predictors for the uptake of the complete schedule of RVV and PCV were region, wealth index and ANC utilization. Accordingly, as borne out in this analysis, children from the Afar region were less likely to be fully vaccinated with RVV (AOR 0.16; 95%-CI 0.04–0.61; *p* < 0.05) compared to children in Addis Ababa. Likewise, children living in Afar or Gambela were 0.10 and 0.25 times less likely to be vaccinated with the complete schedule of PCV, respectively, compared to those in Addis Ababa. There appeared to be a trend that children of mothers from the richer wealth groups were more likely to be vaccinated. Indeed, children from the richer wealth quantile were 1,69 times more likely to receive the complete schedule of RVV (AOR 1.69 95%-CI 1.04–2.75; *p* = 0.036). For PCV vaccination, the trend was similar but not statistically significant. The uptake of RVV and PCV was significantly higher among children born to mothers who had adequate ANC visits (4 or more than 4 visits) compared to those born to mothers who had no ANC visit at all (AOR 2.79 95%-CI 1.73–4.51; *p* < 0.001 and AOR 3.27 95%-CI 2.04–5.23; *p* < 0.001, respectively). Similarly, mothers with inadequate ANC visits (less than 4 visits) were also more likely to have their children vaccinated with the complete schedule of RVV (AOR 1.64 95%-CI 1.06–2.53; *p* < 0.001) or PCV (AOR 2.20 95%-CI 1.43–3.37; *p* = 0.025) than those with no ANC visit at all.

## Discussion

In developing countries, where the economic and public health burden of vaccine-preventable disease is substantial, the potential benefit of including new vaccines in national immunization programmes against diseases not previously covered by the existing programmes is enormous [28]. Working towards achieving high universal coverage is also a key issue in order to optimize the benefits of vaccination programmes. Therefore, policymakers need to closely monitor vaccination coverage and take evidence-based measures to stimulate vaccine uptake and reduce coverage inequalities among different segments of the population. This study aimed to identify the factors associated with the uptake of two newly introduced vaccines, RVV and PCV, in Ethiopia.

It was found that 56 and 49.1% of the Ethiopian children were vaccinated with the complete schedule of RVV and PCV, respectively. The slight difference in coverage between the two vaccines might be attributed to the difference in the number of doses of the vaccines—2 for RVV versus 3 for PCV. Compared to the two-dose RVV, there is a higher possibility of dropout for the three-dose PCV [29]. Slight differences in coverage between the two vaccines have also been noted elsewhere [30]. According to the EDHS report, the coverage also varies between other vaccines included in the routine EPI schedule in Ethiopia [15]. The observed coverage of the two vaccines in Ethiopia is far below the target (>90%) set by the country [10].

There are several factors that can predict childhood immunization coverage [31]. In our analysis, we identified that administrative regions, wealth status, and ANC visits were significantly associated with uptake of the complete schedule of RVV and PCV. With regard to administrative regions, children in the Afar or Gambela regions were less likely to be (fully) vaccinated with RVV or PCV. Likewise, children who live in Afar or Gambela were also less likely to receive the complete schedule of PCV. In Afar and Gambela, significant proportions of the population are pastoralists, commonly moving from one place to another in search of food and water, which makes the provision of health care services challenging including childhood immunization. Moreover, as emerging/developing regions, Afar and Gambela are characterized by fragile health care systems often with poor organization and inadequate infrastructure compared to other regions in the country. This makes it even more difficult to provide vaccination services. Regional variation of immunization coverage within a country has also been documented in several studies from other developing countries [18, 24, 30, 32–34].

Wealth-based health inequality is a major impediment for the realization of universal health-care coverage. In this study, children from the richer wealth quantile were more likely to receive the complete schedule of RVV. Although childhood immunization by itself is free of charge in Ethiopia, there is nevertheless a heavy economic burden associated with childhood vaccination due to travel costs and forgone income because of work loss while seeking immunization services for the children. This finding is in concordance with that in other previous studies [32, 33, 35, 36].

As noted previously [19, 24, 33, 37–39], our study showed that childhood vaccination with RVV and PCV was found to be significantly associated with ANC service utilization. The odds of vaccination with complete schedule RVV and PCV were 2.8 and 3.3 times higher for children whose mothers received adequate ANC (≥4 visit) and 1.6 and 2.2 times higher for children whose mothers received inadequate ANC (< 4 visit), compared to those whose mothers had not made any ANC visit at all. This may be explained by the fact that ANC visits might have improved the mothers’ knowledge and attitude towards the importance of childhood vaccination. In addition, prior experience and interaction during ANC might build trust towards vaccination services, which is important for reducing the vaccine confidence gap in the community.

The strength of the current study is that it is based on nationwide data. However, there are several limitations as well. Firstly, since mothers’ self-report is used as a source of information for the child’s immunization status, there is a possibility of recall bias. However, a study in Tanzania documented a high accuracy and minimal bias due to parental recall during estimation of vaccination coverage compared to card-based data [40]. Secondly, in our study, we focused mainly on the demand side of the determinants and did not assess the effect of the supply side on the uptake of vaccines, because these data are lacking in the EDHS. Clearly, vaccine supply will also have a significant effect on immunization coverage. Lastly, the current study is focusing only on vaccines newly introduced to the country. Despite this limitation, the findings of this study will provide transferable lessons for other vaccine programs within Ethiopia especially for those having the same schedule with vaccines under consideration in our study.

## Conclusions

The overall coverage of RVV and PCV among children between 12 and 23 months of age was suboptimal and below the national target in Ethiopia. Regional variation, wealth status and utilization of ANC were found to be significant predictors for the uptake of the vaccines. Vaccination coverage among children from the Afar and Gambela regions, as well as among those from the poorest sections of the population, was found to be minimal. Hence, this study emphasizes the need for specific interventions, that take regional and wealth status differences into consideration, in order to improve childhood vaccination coverage in the country. Policy makers might consider expanding ANC service utilization among mothers as a potential strategy to improve childhood immunization coverage in Ethiopia.

## Supplementary information

**Additional file 1: Figure S1.** Percentage* of children aged 12–23 months who are fully vaccinated with RVV and PCV in Ethiopia, by a source of information (Vaccination card‡ seen at home, Vaccination record at health facility†, Mother’s report and Any source), Ethiopia DHS 2016.

## Data Availability

Ethiopia Demographic and Health Survey 2016 data was used in this study; it is available for public use upon registration and request to DHS program at https://dhsprogram.com/

## Abbreviations

AOR: Adjusted odds ratio
CIs: Confidence intervals
COR: Crude odds ratio
DTP: Diphtheria-pertussis-tetanus
EDHS: Ethiopian Demographic and Health Survey
EPI: Expanded programme on immunization
GVAP: Global Vaccine Action Plan
PCV: Pneumococcal conjugate vaccine
RED: Reach Every District
RVV: Rotavirus vaccine
SNNP: Southern Nations Nationalities and Peoples
WHO: World Health Organization
