# Supplementary material for: Factors associated with the uptake of newly introduced childhood vaccinations in Ethiopia: the cases of rotavirus and pneumococcal conjugate vaccines
Source: BMC Public Health. 2019 Dec 10;19:1656. doi: 10.1186/s12889-019-8002-8 (PMC6902476; doi:10.1186/s12889-019-8002-8)
Supplement: Supplementary file 1 — Additional file 1: Figure S1. Percentage* of children aged 12–23 months who are fully vaccinated with RVV and PCV in Ethiopia, by a source of information (Vaccination card‡ seen at home, Vaccination record at health facility†, Mother’s report and Any source), Ethiopia DHS 2016. [file 12889_2019_8002_MOESM1_ESM.docx]

**Figure S1:** Percentage* of children aged 12–23 months who are fully vaccinated with RVV and PCV in Ethiopia, by a source of information (Vaccination card^‡^ seen at home, Vaccination record at health facility^†^, Mother’s report and Any source), Ethiopia DHS 2016.

*****shows the proportion within the group

**‡**also includes booklet or other home-based record

**†**vaccination information was obtained from health facility record for children who did not have vaccination card during home visit but received at least one vaccination at health facility. This is only for children whose mother give a consent to see the data at health facility.
